# Reduced circulating interleukin 35 is associated with enhanced peripheral T cell function in primary biliary cholangitis

**DOI:** 10.17305/bjbms.2022.8147

**Published:** 2023-03-16

**Authors:** Siqi Liu, Qian Zhang, Mengyao Zhang, Xuejing Zhong, Wudong Wang, Lishuang Wang, Zhenjing Jin

**Affiliations:** 1 Digestive Disease Center, Department of Hepatopancreatobiliary Medicine, The Second Hospital, Jilin University, Changchun, Jilin Province, China

**Keywords:** Primary biliary cholangitis (PBC), interleukin 35 (IL-35), T lymphocytes

## Abstract

Interleukin 35 (IL-35) mediates immunosuppression of T cells in autoimmune diseases. T cells play an important role in primary biliary cholangitis (PBC) with incompletely elucidated pathogenesis. Thus, we aimed to investigate the role of IL-35 regulation on T cells in PBC patients. Fifty-one PBC patients and 28 controls were enrolled in this study. Plasma IL-35 level was measured. Purified peripheral CD4^+^ and CD8^+^ T cells were stimulated with exogenous IL-35 to investigate their functional phenotypes. IL-35-treated CD8^+^ T cells were cultured with human intrahepatic biliary epithelial cell line to determine the cytotoxicity of CD8^+^ T cells from PBC patients. Plasma IL-35 concentration was lower in PBC patients and negatively correlated with alkaline phosphatase. CD4^+^ T cells from PBC patients exhibited elevated transcription factor expressions and cytokine secretion, whereas CD8^+^ T cells produced increased cytotoxic molecules and cytokines. In vitro IL-35 stimulation suppressed the production of IL-17 and IL-22 by CD4^+^ T cells from PBC patients. CD8^+^ T cells treated with IL-35 mediated reduced target cell death in the direct contact co-culture system in PBC patients. This process was accompanied by reduced production of cytotoxic molecules and cytokines and increased expressions of immune checkpoint receptors in CD8^+^ T cells. Reduced circulating IL-35 might be insufficient to suppress T cell function, leading to the immune dysregulation in PBC patients.

## Introduction

Primary biliary cholangitis (PBC), formerly known as primary biliary cirrhosis, is an autoimmune disease which predominantly affects middle-aged females [1]. The characteristic of PBC is the chronic progressive destruction of small intrahepatic bile ducts, leading to cholestasis, portal inflammation, fibrosis, and biliary cirrhosis [1–3]. The serological hallmark of PBC is the presence of anti-mitochondrial autoantibodies (AMAs) [1]. Although the etiology of PBC is still not fully elucidated, the pathogenesis of PBC is closely associated with the loss of immune tolerance against mitochondrial antigens, resulting in the immune-mediated biliary damage [1, 4]. Previous report showed that the numbers of peripheral T helper (Th) cells were elevated, and the activations of peripheral Th cells correlated with the severity of PBC, indicating the involvement of CD4^+^ Th cells in the pathogenesis of PBC [5]. Liver-infiltrating terminally differentiated CD8^+^ T cells revealed elevated cytokine production ability and cytotoxicity in a PBC mouse model, suggesting the key role of hepatic CD8^+^ T cells in the pathogenesis of PBC [6]. However, the regulation of T cells in PBC patients is still not completely understood.

Interleukin 35 (IL-35) is a newly identified IL-12 cytokine family member and consists of two heterodimeric subunits, Epstein-Barr virus-induced gene 3 (IL-27β chain) and IL-12α chain p35. IL-35 is released by CD4^+^FoxP3^+^ regulatory T cells, regulatory B cells, and regulatory CD8^+^ T cells [7, 8]. IL-35 receptor is also a heterodimer, including IL-12 receptor β2 (IL-12Rβ2) and gp130, which is mainly expressed on immune cells, such as T cells, B cells, and monocytes [9]. Thus, signaling through IL-35/IL-35 receptor pathways always regulates immune cell activity to mediate immunosuppression by inhibition of effector T cell function and enhancement of regulatory cell activity [10]. Our previous studies demonstrated that elevated IL-35 contributed to immune cell dysregulation in various liver diseases, including chronic viral hepatitis [11–13], liver cirrhosis [14], acute-on-chronic liver failure [15], and hepatocellular carcinoma [16–18]. Abnormal expression of IL-35 was also found in several inflammatory autoimmune diseases, leading to the onset and development of these diseases [19, 20]. PBC is an organ-specific autoimmune disease [1]. Li et al. revealed lower plasma IL-35 level in PBC patients, and IL-35 was capable of promoting the inhibitory function of regulatory T cells in PBC patients [21]. However, few studies have focused on the IL-35 regulation of T cells in PBC patients. Accordingly, we examined the correlation between IL-35 and clinical index in PBC patients. Subsequently, peripheral CD4^+^ and CD8^+^ T cell activity from PBC patients was functionally analyzed in response to exogenous IL-35 stimulation in vitro.

## Materials and methods

### Patients and controls

This study complied with all relevant national regulations, institutional policies and was in accordance with the tenets of the Helsinki Declaration (as revised in 2013) and has been approved by the Ethics Committee of the Second Hospital of Jilin University (IRB No. 26/06/17 on 07.06.2017). Written informed consents were obtained from all individuals included in this study. The sample size numbers were calculated by Clinical Research Sample Size Calculator. Fifty-one PBC patients and 28 sex- and age-matched healthy individuals were included between July 2020 and July 2021. The diagnosis of PBC was based on established criteria [22]. All patients were treatment-naive and positive for anti-AMAs. Individuals who were coinfected with hepatitis virus, human immunodeficiency virus, or afflicted with alcoholic cirrhosis, autoimmune hepatitis, primary sclerosing cholangitis, other autoimmune diseases, or cancers were excluded from this study. The clinical characteristics of all studied subjects are listed in Table 1.

**Table 1:** Clinical characteristics of studied subjects

	**Controls**	**PBC patients**
Number	28	51
Sex (male/female)	7/21	8/43
Age, median, IQR (years)	46 (22, 60)	38 (25, 51)
Alanine aminotransferase, median, IQR (IU/L)	26 (20, 34)	62 (48, 112)
Aspartate aminotransferase, median, IQR (IU/L)	20 (14, 29)	122 (71, 209)
Total bile acid, median, IQR (µmol/L)	7.8 (4.1, 20.1)	70.0 (58.0, 73.7)
Alkaline phosphatase, median, IQR (IU/L)	54 (41, 67)	124 (86, 171)

PBC: Primary biliary cholangitis; IQR: Interquartile range.

### Plasma and cell sorting

Ethylene diamine tetraacetic acid anti-coagulant peripheral blood samples were collected from all subjects. Plasma was harvested by centrifugation at 1000×*g* for 10 min. Peripheral blood mononuclear cells (PBMCs) were isolated by density gradient centrifugation using Ficoll-Hypaque (Sigma-Aldrich, St Louis, MO, USA). CD4^+^ T cells were purified using Human CD4 MicroBeads (Miltenyi, Bergisch Gladbach, Germany; Catalog# 130-045-101), while CD8^+^ T cells were sorted using Human CD8^+^ T cell Isolation Kit (Miltenyi; Catalog# 130-117-027) as previously described [14].

### Cell stimulation and culture

The regular bottom cell culture plates were coated with anti-human CD3 epsilon antibody (R&D System, Minneapolis, MN, USA; Catalog# MAB100-500, Clone UCHT1; final concentration: 1 µg/mL) and anti-human CD28 antibody (R&D System; Catalog# MAB342-500, Clone 37407; final concentration: 1 µg/mL) overnight at 4 ^∘^C as previously described [14]. Sorted CD4^+^ and CD8^+^ T cells were then added into coated plates, and cultured with phytohaemagglutinin (PHA) (final concentration: 1 µg/mL) in the presence or absence of recombinant human IL-35 (Peprotech, Rocky Hill, NJ, USA; Catalog# 200–37; final concentration: 1 ng/mL) as previously used [11–18]. The human intrahepatic biliary epithelial cells (HIBECs) (ScienCell, San Diego, CA, USA) were stably transfected with pcDNA3.1-HLA-A*0201 plasmid and were incubated in RPMI 1640 supplemented by 10% fetal bovine serum, 100 U/L penicillin, and 0.1 mg/mL streptomycin in the presence of G418 antibiotic (final concentration: 6.5 mg/mL) at 37 ^∘^C under 5% CO_2_ conditions. In certain experiments, CD8^+^ T cells, which were sorted from 9 HLA-A*02 restricted PBC patients, were stimulated with recombinant human IL-35 for 24 h in the presence of anti-CD3/CD28 antibody. 10^4^ of IL-35 stimulated CD8^+^ T cells from HLA-A*02 restricted PBC patients were co-cultured in direct contact or indirect contact with 5 × 10^4^ of pcDNA3.1-HLA-A*0201 stably transfected HIBECs in the presence of anti-human CD3/CD28 antibody as previously described [12, 14, 16]. Cells and supernatants were harvested for further experiments 48 h post co-culture.

### Enzyme-linked immunosorbent assay

To analyze IL-35 concentration in the plasma and cytokine concentrations in the cultured supernatants, cytokines and cytotoxic molecules levels were measured using commercial enzyme-linked immunosorbent assay (ELISA) kits (CUSABIO, Wuhan, Hubei Province, China), including human IL-35 ELISA kit (Catalog# CSB-E13126h, sensitivity 15.6 pg/mL), human interferon γ (IFN-γ) ELISA kit (Catalog# CSB-E04577h, sensitivity 1.56 pg/mL), human IL-9 ELISA kit (Catalog# CSB-E04642h, sensitivity 3.9 pg/mL), human IL-17 ELISA kit (Catalog# CSB-E12819h, sensitivity 15.6 pg/mL), human IL-22 ELISA kit (Catalog# CSB-E13418h, sensitivity 19.5 pg/mL), human tumor necrosis factor α (TNF-α) ELISA kit (Catalog# CSB-E04740h, sensitivity 1.95 pg/mL), human perforin-1 ELISA kit (Catalog# CSB-E09313h, sensitivity 0.078 ng/mL), human granzyme B ELISA kit (Catalog# CSB-E08718h, sensitivity 0.39 pg/mL), and human granulysin ELISA kit (Catalog# CSB-E09936h).

### Flow cytometry

The absolute T cell counts and immune-checkpoint receptors expression on CD8^+^ T cells were analyzed by flow cytometry, which was performed as previously described [14]. CD3^+^, CD4^+^, and CD8^+^ T cell counts were analyzed using BD Tritest CD4 fluorescein isothiocyanate (FITC)/CD8 phycoerythrin (PE)/CD3 peridinin chlorophyll protein (PerCP) (BD Tritest, San Jose, CA, USA; Catalog# 340298) in a BD TruCount tube (BD Tritest). Harvested CD8^+^ T cells were stained with allophycocyanin Cy7 mouse anti-human CD8 (BD Pharmingen, San Jose, CA, USA; Catalog# 557834, Clone SK1), FITC mouse anti-human programmed death-1 (PD-1) (BD Pharmingen; Catalog# 557860, Clone MIH4), and PE mouse anti-human cytotoxic T-lymphocyte-associated protein-4 (CTLA-4) (BD Pharmingen; Catalog# 557301, Clone BNI3). Isotype controls were used to enable precise compensation and confirm antibody specificity. Data were acquired using a FACS Calibur flow cytometer (BD Biosciences Immunocytometry Systems, San Jose, CA, USA) and were analyzed using FlowJo software Version 10 (TreeStar, Ashland, OR, USA).

**Figure 1.**
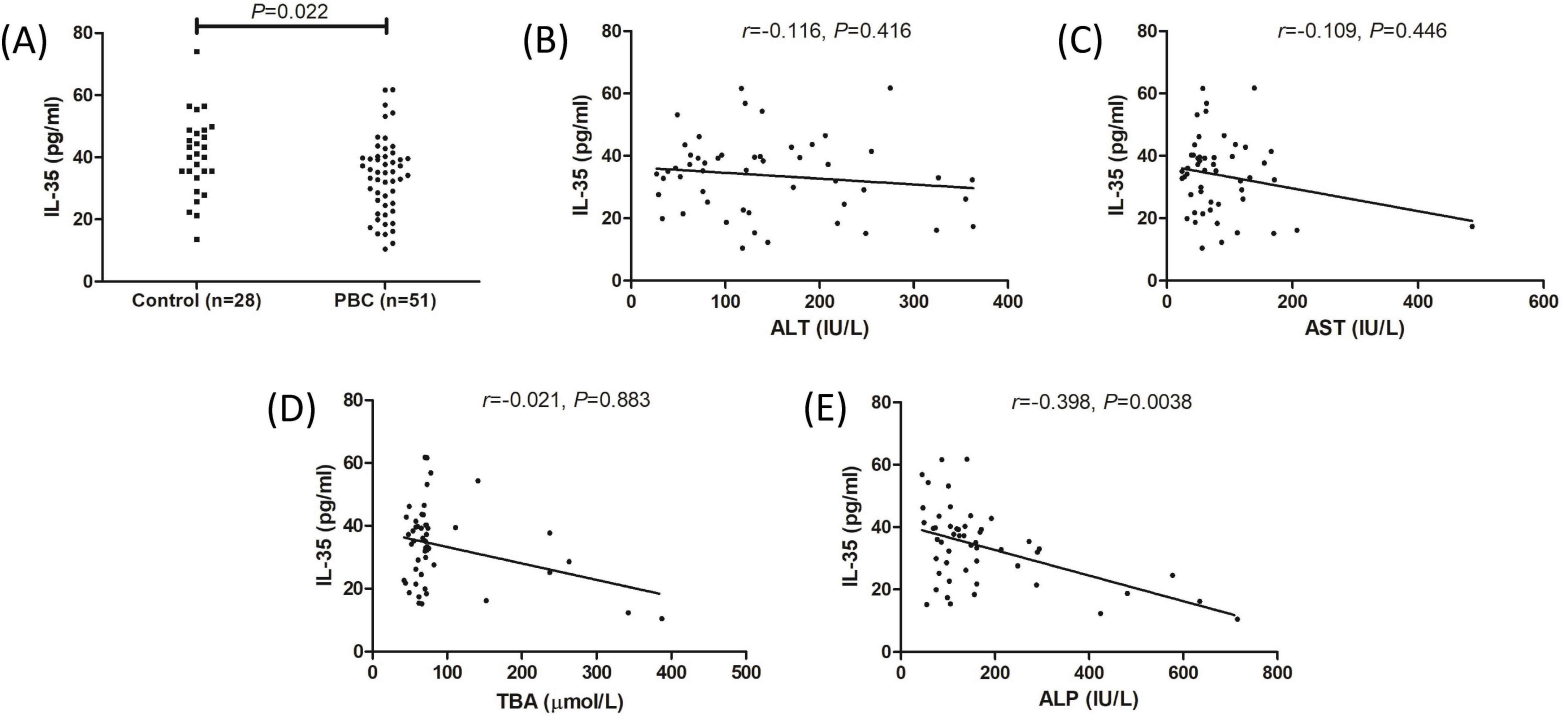
**Plasma IL-35 expression in controls and PBC patients**. The concentration of IL-35 in plasma was measured by ELISA in controls (*n* ═ 28) and PBC patients (*n* ═ 51). (A) Plasma IL-35 level was lower in PBC patients compared with controls. The individual level for each subject was shown. Significance was assessed using Student’s t test. Plasma IL-35 level did not correlate with (B) ALT level, (C) AST level, and (D) TBA level in PBC patients. (E) Plasma IL-35 level negatively correlated with ALP level in PBC patients. Correlation analysis was made using Spearman correlation analysis. PBC: Primary biliary cholangitis; IL: Interleukin; ALT: Alanine aminotransferase; AST: Aspartate aminotransferase; TBA: Total bile acid; ALP: Alkaline phosphatase.

### Real-time reverse transcription-polymerase chain reaction

mRNA relative levels corresponding to IL-35 receptor subunits and transcription factors were analyzed by real-time reverse transcription polymerase chain reaction (RT-PCR), which was performed as previously described [11]. Briefly, total RNA was extracted using TRIzol reagent (Invitrogen, Carlsbad, CA, USA; Catalog# 15596-018). First-strand cDNA was synthesized with oligo(dT) primer using PrimeScript RT Master Mix (TaKaRa, Beijing, China; Catalog# RR036B). Real-time PCR was performed using TB Green *Premix Ex Taq* (TaKaRa; Catalog# RR42WR). The relative gene expressions were quantified using ABI7500 System Sequence Detection Software (Applied Biosystems, Foster, CA, USA). The primers for IL-35 receptor subunits, including IL-12Rβ2 and gp130, were purchased from BioRad (Hercules, CA, USA; Catalog# qHsaCID0006511 and qHsaCID0007540). Other primer sequences were obtained from previously published literature [11, 23].

### Cytotoxicity of HIBECs

To analyze the cytotoxicity of HIBECs, lactate dehydrogenase (LDH) level in the supernatants was measured at the end of the incubation period using LDH Cytotoxicity Assay Kit (Beyotime, Wuhan, Hubei Province, China; Catalog# C0016) as previously described [14]. The LDH level in the cultured HIBECs was determined to be “low-level control,” whereas the LDH level in Triton X100-treated HIBECs was determined to be “high-level control.” The percentage of HIBECs death was calculated using the following equation:

(experimental value − low level control)/(high level control − low level control)×100% [14].

### Statistical analysis

Data were analyzed using SPSS23.0 for Windows (IBM Corp, Armonk, NY, USA). The Shapiro-Wilk test was used for the normal distribution assay. Data following normal distribution were presented as mean ± standard deviation. Statistical analyses were performed using Student’s t test or paired t test. Pearson correlation analyses were used for correlation analysis for normal distribution data. Data following skewed distribution were presented as median and interquartile range (IQR). Statistical analyses were performed using Mann–Whitney U test or Wilcoxon matched pairs test. Spearman correlation analyses were used for correlation analysis for skewed distribution data. A *P* value less than 0.05 was considered as statistical difference.

## Results

### Plasma IL-35 level was reduced in PBC patients

**Figure 2.**
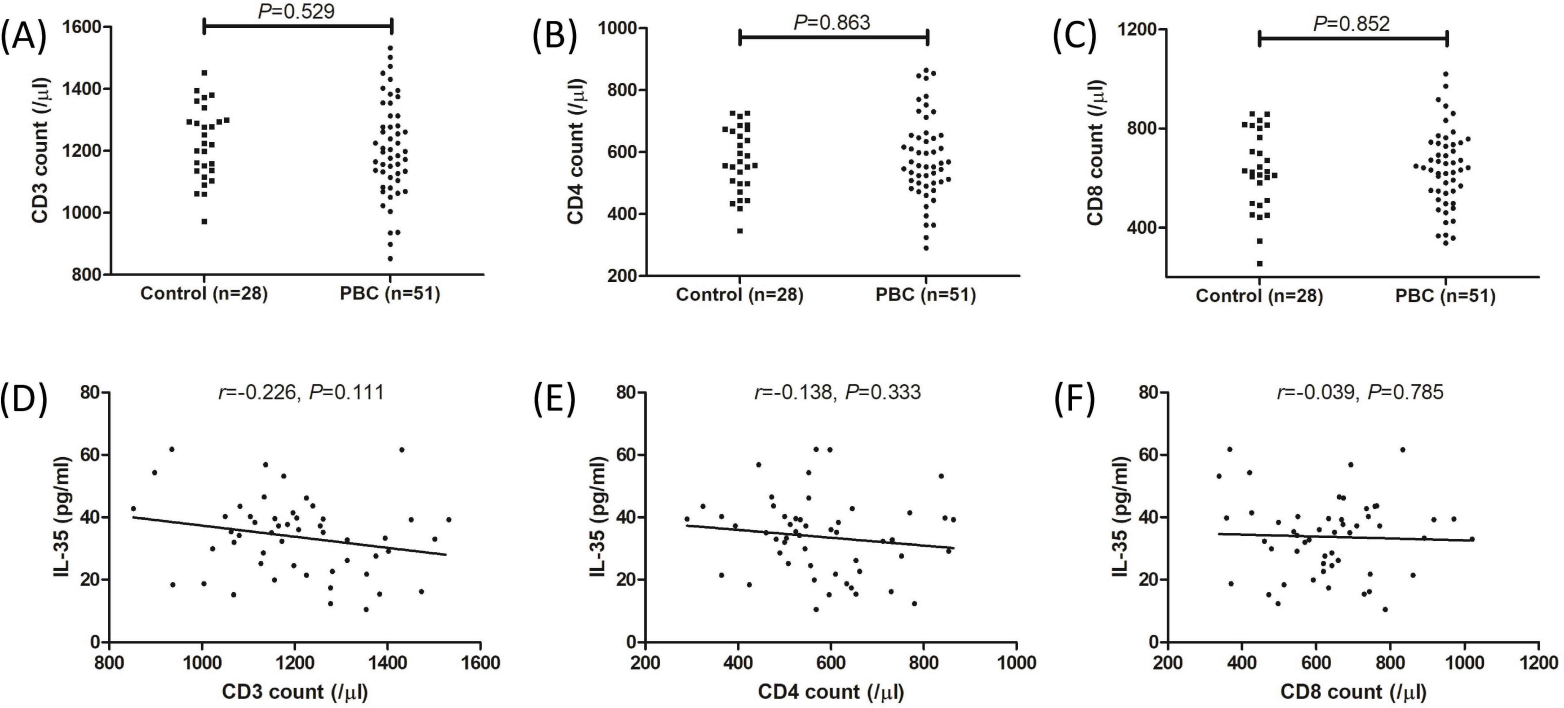
**Peripheral T cell counts in controls and PBC patients**. Peripheral T cell counts were measured by flow cytometry in controls (*n* ═ 28) and PBC patients (*n* ═ 51). There were no remarkable differences in (A) CD3^+^ T cell count, (B) CD4^+^ T cell count, and (C) CD8^+^ T cell count between controls and PBC patients. The individual level for each subject was shown. Significance was assessed using Student’s t test. Plasma IL-35 level did not correlate with (D) CD3^+^ T cell count, (E) CD4^+^ T cell count, and (F) CD8^+^ T cell count in PBC patients. Correlation analysis was made using Pearson correlation analysis. PBC: Primary biliary cholangitis; IL: Interleukin.

We first examined plasma IL-35 concentration by ELISA. Plasma IL-35 concentration was significantly lower in PBC patients compared to controls (33.72 ± 12.27 pg/mL vs 40.55 ± 12.65 pg/mL; *P* ═ 0.022, Figure 1A). There were no statistical correlations between plasma IL-35 level and alanine aminotransferase (ALT) (*r* ═ –0.116, *P* ═ 0.416, Figure 1B), aspartate aminotransferase (AST) (*r* ═ –0.109, *P* ═ 0.446, Figure 1C), or total bile acid (TBA) (*r* ═ –0.021, *P* ═ 0.883, Figure 1D). Plasma IL-35 level was negatively correlated with alkaline phosphatase (ALP) (*r* ═ –0.398, *P* ═ 0.0038, Figure 1E). Peripheral T cell counts were measured by flow cytometry. There were no remarkable differences in either CD3^+^ T cell count (1224 ± 118.2/µL vs 1203 ± 155.5/µL; *P* ═ 0.529, Figure 2A), CD4^+^ T cell count (572.9 ± 104.2/µL vs 578.0 ± 135.2/µL; *P* ═ 0.863, Figure 2B), nor CD8^+^ T cell count (630.0 ± 159.3/µL vs 636.9 ± 155.8/µL; *P* ═ 0.852, Figure 2C) between controls and PBC patients. Plasma IL-35 level in PBC patients did not correlate with CD3^+^ T cell count (*r* ═ –0.226, *P* ═ 0.111, Figure 2D), CD4^+^ T cell count (*r* ═ –0.138, *P* ═ 0.333, Figure 2E), or CD8^+^ T cell count (*r* ═ –0.039, *P* ═ 0.785, Figure 2F).

### Peripheral CD4+ and CD8+ T cells presented stronger inflammatory and cytotoxic phenotype in PBC patients

**Figure 3.**
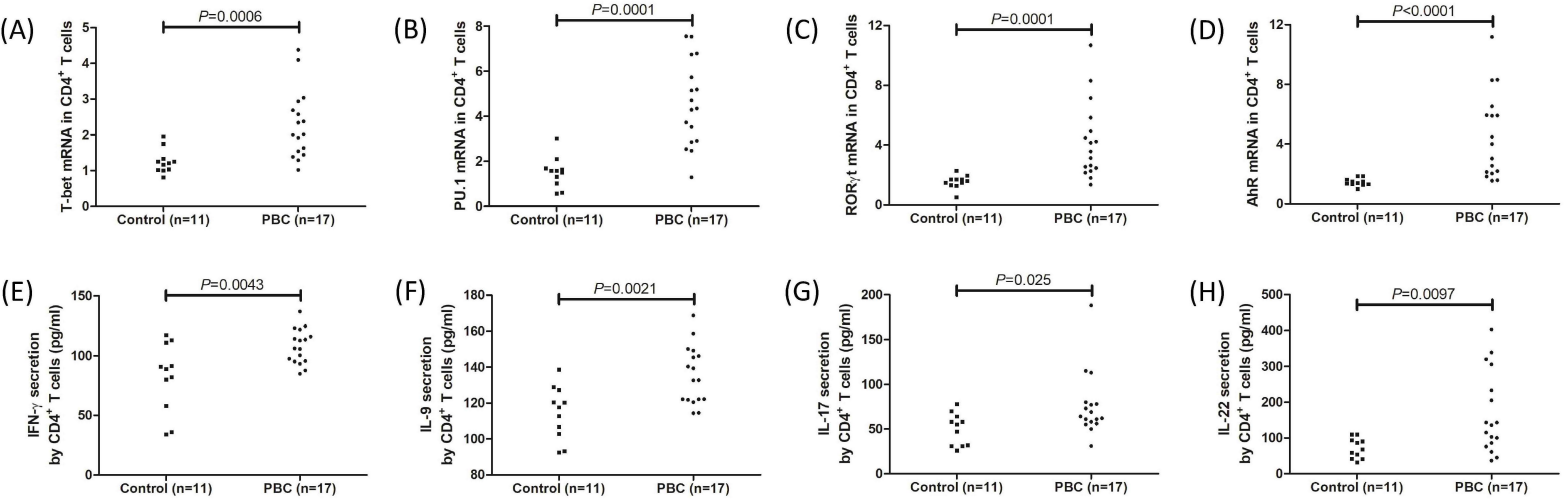
**Transcription factor mRNA expression and cytokine secretion in CD4^+^ T cells in controls and PBC patients.** Peripheral CD4^+^ T cells were sorted from controls (*n* ═ 11) and PBC patients (*n* ═ 17) and cultured with anti-CD3/CD28 and PHA for 12 h. Cells were harvested for real-time RT-PCR, while supernatants were harvested for ELISA analysis. mRNA relative levels of (A) Th1 transcription factor T-bet, (B) Th9 transcription factor PU.1, (C) Th17 transcription factor RORγt, and (D) Th22 transcription AhR were elevated in CD4^+^ T cells from PBC patients when compared with those from controls. The expressions of (E) Th1-related cytokine IFN-γ, (F) Th9-related cytokine IL-9, (G) Th17-related cytokine IL-17, and (H) Th22-related cytokine IL-22 were also increased in the cultured supernatants of CD4^+^ T cells from PBC patients when compared with those from controls. The individual level for each subject was shown. Significance was assessed using Student’s t test or Mann–Whitney U test. PBC: Primary biliary cholangitis; IL: Interleukin; RORγt: Retinoid-related orphan receptor γt; AhR: Aryl hydrocarbon receptor; RT-PCR: Reverse transcription–polymerase chain reaction.

**Figure 4.**
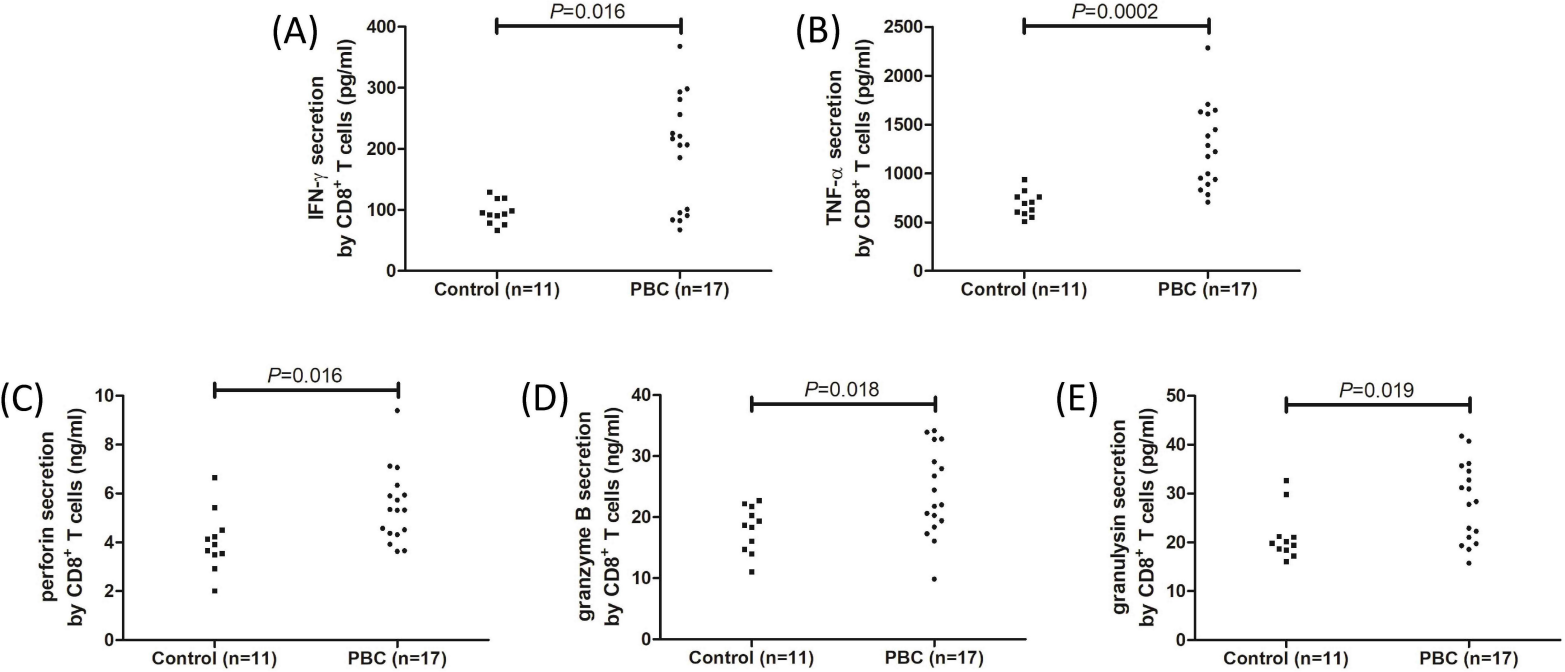
**Cytokine and cytotoxic molecule secretion by CD8^+^ T cells in controls and PBC patients.** Peripheral CD8^+^ T cells were sorted from controls (*n* ═ 11) and PBC patients (*n* ═ 17) and cultured with anti-CD3/CD28 and PHA for 12 h. Supernatants were harvested for ELISA analysis. The expressions of (A) IFN-γ, (B) TNF-α, (C) perforin, (D) granzyme B, and (E) granulysin were increased in the cultured supernatants of CD8^+^ T cells from PBC patients when compared with those from controls. The individual level for each subject was shown. Significance was assessed using Student’s t test or Mann–Whitney U test. PBC: Primary biliary cholangitis; IFN-γ: Interferon γ; TNF-α: Tumor necrosis factor α; IL: Interleukin; RT-PCR: Reverse transcription–polymerase chain reaction; PHA: Phytohaemagglutinin.

Peripheral CD4^+^ and CD8^+^ T cells were sorted from 11 controls and 17 PBC patients and were cultured with anti-CD3/CD28 antibody and PHA for 12 h. Compared with those from controls, CD4^+^ T cells from PBC patients had elevated mRNA relative levels corresponding to Th1 transcription factor T-bet (2.02 [IQR 1.49, 2.82] vs 1.21 [IQR 1.02, 1.33]; *P* ═ 0.0006, Figure 3A), Th9 transcription factor PU.1 (4.35 [IQR 2.88, 6.24] vs 1.58 [IQR 1.02, 1.69]; *P* ═ 0.0001, Figure 3), Th17 transcription factor retinoid-related orphan receptor γt (RORγt) (3.58 [IQR 2.37, 5.40] vs 1.60 [IQR 1.33, 1.71]; *P* ═ 0.0001, Figure 3C), and Th22 transcription factor aryl hydrocarbon receptor (AhR) (4.00 [IQR 2.09, 6.24] vs 1.42 [IQR 1.33, 1.63]; *P*< 0.0001, Figure 3D). Similarly, the concentrations of secreting cytokines by CD4^+^ T cells were higher in the supernatants of cultured CD4^+^ T cells from PBC patients when compared with controls, including Th1-related cytokine IFN-γ (107.7 ± 14.48 pg/mL vs 82.07 ± 28.76 pg/mL; *P* ═ 0.0043, Figure 3E), Th9-related cytokine IL-9 (135.4 ± 16.11 pg/mL vs 114.7 ± 14.72 pg/mL; *P* ═ 0.0021, Figure 3F), Th17-related cytokine IL-17 (64.94 [IQR 57.50, 79.61] pg/mL vs 55.00 [IQR 31.79, 64.36] pg/mL; *P* ═ 0.025, Figure 3G), and Th22-related cytokine IL-22 (136.7 [IQR 81.32, 269.4] pg/mL vs 68.64 [IQR 42.15, 94.49] pg/mL; *P* ═ 0.0097, Figure 3H). CD8^+^ T cells from PBC patients secreted the increased amount of proinflammatory cytokines compared with controls, including IFN-γ (206.8 [IQR 93.09, 268.9] pg/mL vs 93.72 [IQR 78.84, 118.7] pg/mL; *P* ═ 0.016, Figure 4A) and TNF-α (1264 ± 421.7 pg/mL vs 687.4 ± 128.0 pg/mL; *P* ═ 0.0002, Figure 4B), as well as cytotoxic molecules, including perforin (5.44 ± 1.49 ng/mL vs 4.05 ± 1.23 ng/mL; *P* ═ 0.016, Figure 4C), granzyme B (23.98 ± 7.08 pg/mL vs 18.11 ± 3.75 pg/mL; *P* ═ 0.018, Figure 4D), and granulysin (28.21 ± 8.11 pg/mL vs 21.33 ± 5.17 pg/mL; *P* ═ 0.019, Figure 4E).

### In vitro recombinant human IL-35 stimulation inhibited IL-17 and IL-22 production by CD4+ T cells from PBC patients

**Figure 5.**
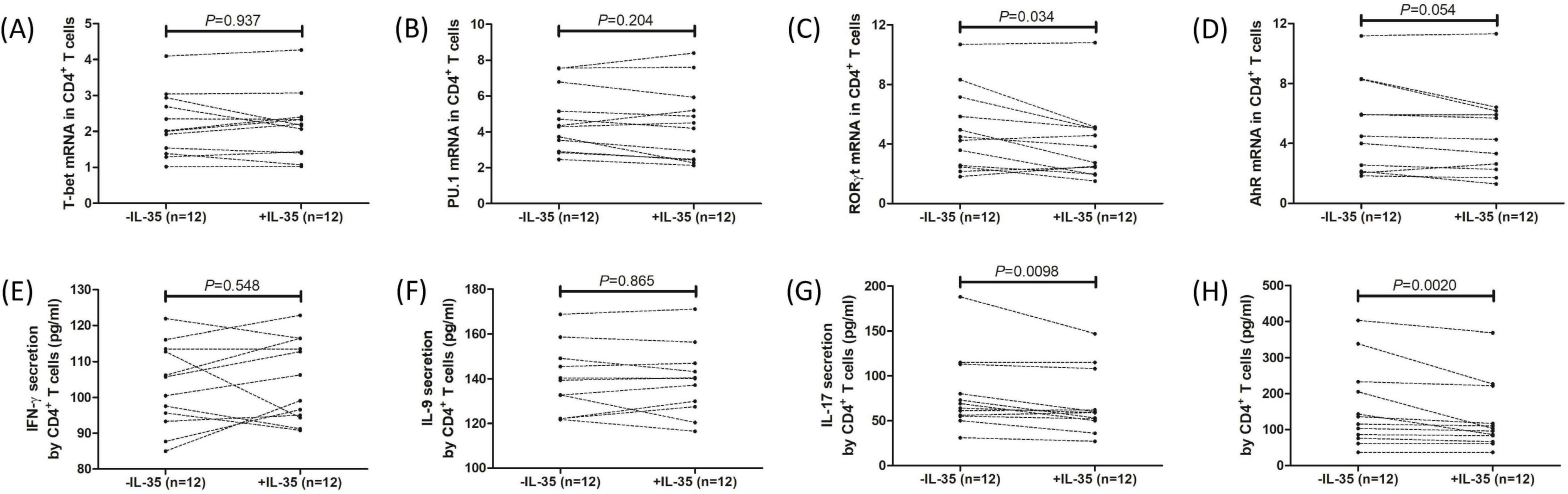
**Changes in transcription factor mRNA expression and cytokine secretion in CD4^+^ T cells from PBC patients in response to recombinant human IL-35 stimulation in vitro.** Peripheral CD4^+^ T cells were sorted from PBC patients (*n* ═ 12) and cultured with anti-CD3/CD28 and PHA in the presence or absence of recombinant human IL-35 for 12 h. Cells were harvested for real-time RT-PCR, while supernatants were harvested for ELISA analysis. mRNA relative levels of (A) *T-bet* and (B) *PU.1* in CD4^+^ T cells were not affected by recombinant human IL-35 stimulation. (C) IL-35 stimulation reduced *RORγt* mRNA relative level in CD4^+^ T cells. (D) *AhR* mRNA relative level in CD4^+^ T cells was slightly decreased in response to IL-35 stimulation, but this difference failed to achieve statistical significance. (E) IFN-γ and (F) IL-9 levels in cultured supernatants were not affected by recombinant human IL-35 stimulation. (G) IL-17 and (F) IL-22 levels in cultured supernatants were notably downregulated following IL-35 stimulation. The individual level for each subject was shown. Significance was assessed using paired t test or Wilcoxon matched pairs test. PBC: Primary biliary cholangitis; IFN-γ: Interferon γ; RORγt: Retinoid-related orphan receptor γt; AhR: Aryl hydrocarbon receptor; IL: Interleukin; RT-PCR: Reverse transcription–polymerase chain reaction.

There were no significant differences in IL-35 receptor subunits (IL-12Rβ2 and gp130) in CD4^+^ T cells between controls and PBC patients (Figure S1A and S1B). Peripheral CD4^+^ T cells, which were sorted from 12 PBC patients, were cultured with anti-CD3/CD28 antibody and PHA in the presence or absence of recombinant human IL-35 for 12 h. Recombinant human IL-35 stimulation did not affect *T-bet* and *U.1* mRNA relative levels (Figure 5A and 5B) or IFN-γ and L-9 secretion (Figure 5E and 5F) in CD4^+^ T cells from PBC patients. Exogenous IL-35 stimulation reduced *RORγt* mRNA relative level (3.28 [IQR 2.09, 5.06] vs 4.36 [IQR 2.49, 6.83]; *P* ═ 0.034, Figure 5C) and IL-17 production (59.00 [IQR 50.43, 96.23] pg/mL vs 66.58 [IQR 55.85, 104.8] pg/mL; *P* ═ 0.0098, Figure 5G) in CD4^+^ T cells from PBC patients. Similarly, IL-35 also notably downregulated IL-22 secretion by CD4^+^ T cells (99.54 [IQR 70.90, 195.4] pg/mL vs 125.4 [IQR 78.56, 226.8] pg/mL; *P* ═ 0.0020, Figure 5G). *AhR* mRNA relative level in CD4^+^ T cells was also slightly reduced in response to IL-35 stimulation, but this difference failed to achieve statistical significance (4.99 [IQR 2.36, 6.12] vs 5.19 [IQR 2.24, 7.70]; *P* ═ 0.054, Figure 5D).

### In vitro recombinant IL-35 stimulation suppressed the cytotoxicity of CD8+ T cells from PBC patients

**Figure 6.**
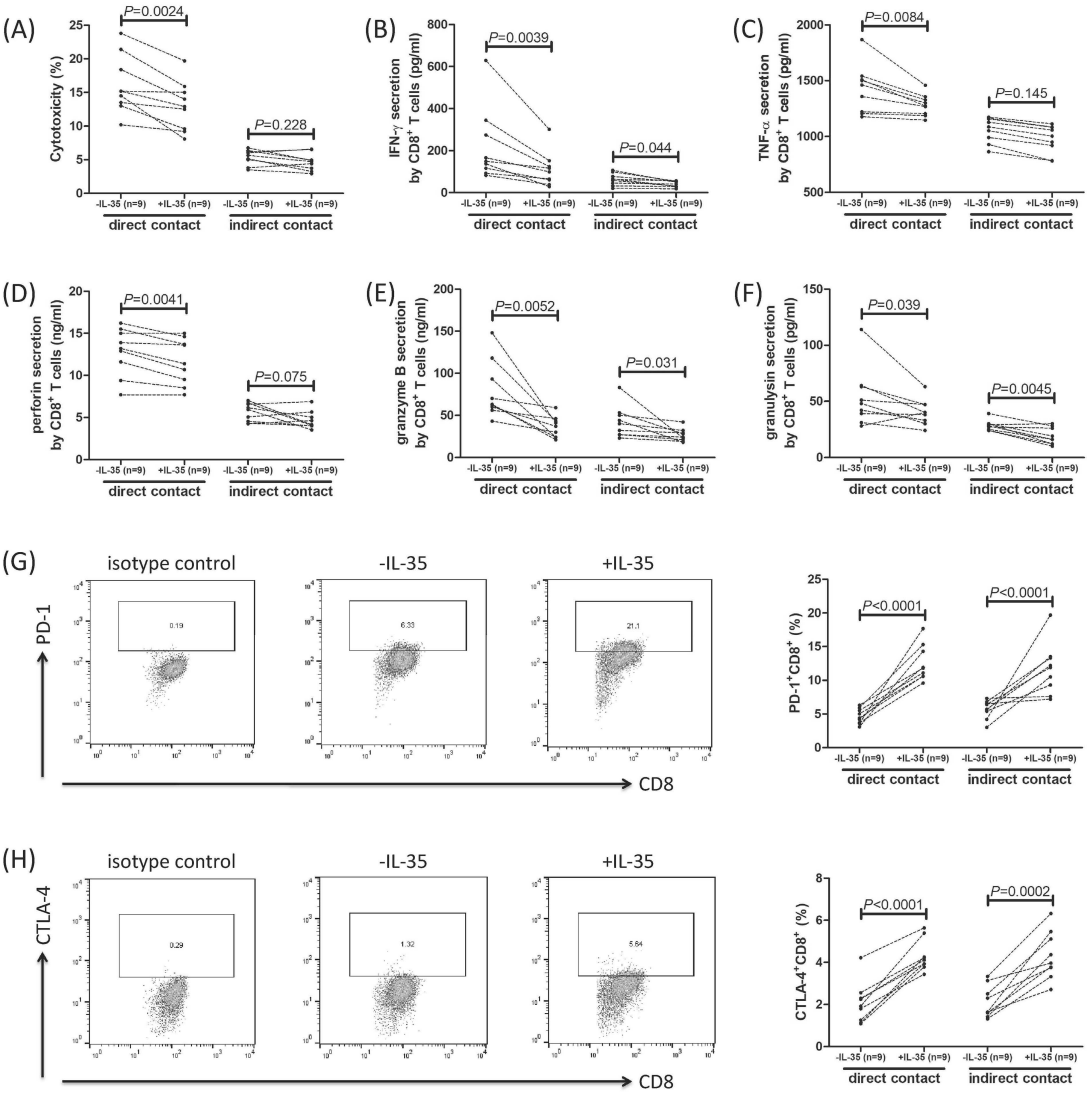
**Changes in cytotoxicity of CD8^+^ T cells, cytokines and cytotoxic molecules secretion, and immune checkpoint receptors expression in CD8^+^ T cells from PBC patients in response to recombinant human IL-35 stimulation in vitro.** CD8^+^ T cells were sorted from HLA-A*02 restricted PBC patients (*n* ═ 9) and were stimulated with recombinant human IL-35 for 24 h in the presence of anti-CD3/CD28 antibody. Cells were washed twice after stimulation. 10^4^ of IL-35 stimulated CD8^+^ T cells were co-cultured in direct or indirect contact with 5 × 10^4^ of pcDNA3.1-HLA-A*0201 transfected HIBECs in the presence of anti-human CD3/CD28 antibody for 48 h. Supernatants were harvested, and percentage of HIBECs death was measured by LDH release, while cytokine production was measured by ELISA. (A) Percentage of HIBECs death in direct and indirect contact co-culture system in the presence or absence of IL-35 stimulation. (B) IFN-γ, (C) TNF-α, (D) perforin, (E) granzyme B, and (F) granulysin levels in the supernatants in direct and indirect contact co-culture system in the presence or absence of IL-35 stimulation. (G) PD-1^+^CD8^+^ and (H) CTLA-4^+^CD8^+^ cell percentages were accessed by flow cytometry and compared between cells in the presence or absence of IL-35 stimulation. The individual level for each subject was shown. Significance was assessed using paired t test or Wilcoxon matched pairs test. PBC: Primary biliary cholangitis; HIBEC: Human intrahepatic biliary epithelial cells; IFN-γ: Interferon γ; TNF-α: Tumor necrosis factor α; IL: Interleukin; LDH: Lactate dehydrogenase.

There were no significant differences in IL-35 receptor subunits (IL-12Rβ2 and gp130) in CD8^+^ T cells between controls and PBC patients (Figure S1C and S1D). In direct contact co-culture system, IL-35-stimulated CD8^+^ T cells induced lower percentage of HIBECs death when compared with unstimulated CD8^+^ T cells (12.99 ± 3.68% vs 16.13 ± 4.30%; *P* ═ 0.0024, Figure 6A). Similarly, IL-35-stimulated CD8^+^ T cells secreted lower levels of IFN-γ (*P* ═ 0.0039, Figure 6B), TNF-α (*P* ═ 0.0084, Figure 6C), perforin (*P* ═ 0.0041, Figure 6D), granzyme B (*P* ═ 0.0052, Figure 6E), and granulysin (*P* ═ 0.039, Figure 6F). However, there was no significant difference in induced HIBECs death between CD8^+^ T cells with and without IL-35 stimulation in indirect contact co-culture system (4.67 ± 1.28% vs 5.38 ± 1.12%; *P* ═ 0.228, Figure 6A). IFN-γ (*P* ═ 0.044, Figure 6B), granzyme B (*P* ═ 0.031, Figure 6E), and granulysin (*P* ═ 0.045, Figure 6F) secretion was lower in IL-35-treated CD8^+^ T cells in indirect contact co-culture system, but IL-35 stimulation did not affect TNF-α (*P* ═ 0.145, Figure 6C) or perforin (*P* ═ 0.075, Figure 6D) production in indirect contact co-culture system. The expression of PD-1 and CTLA-4 was determined in CD8^+^ T cells with and without IL-35 stimulation. Representative flow dots analyses are shown in Figure 6G and 6H. The percentage of CD8^+^ T cells expressing PD-1 and CTLA-4 was notably increased in response to IL-35 stimulation in both direct contact and indirect contact co-culture systems (*P* < 0.0001, Figure 6G and 6H).

## Discussion

In this study, we observed that circulating IL-35 level was significantly lower in PBC patients, and negatively correlated with ALP. Peripheral CD4^+^ and CD8^+^ T cells revealed stronger inflammatory and cytotoxic phenotype in PBC patients, exhibiting increased cytokines secretions and higher cytotoxic molecules productions. Although plasma IL-35 level did not correlate with absolute T cell counts, in vitro exogenous IL-35 stimulation suppressed T cell activity in PBC patients, which presented as inhibiting of IL-17/IL-22 production by CD4^+^ T cells and dampening cytotoxicity of CD8^+^ T cells. The current finding indicated an immunosuppressive property of IL-35 in PBC. Insufficient IL-35 expression might be closely associated with enhanced T cell function, leading to immune dysfunction in PBC patients.

Cytokine-mediated immune response plays a crucial role in the pathogenesis of varieties of autoimmune diseases. IL-35 was implicated in the pathogenesis of systemic lupus erythematosus, rheumatoid arthritis (RA), multiple sclerosis, psoriasis, autoimmune hepatitis, etc. [24]. IL-35 level and IL-35-secreting cells were found to be decreased in patients with systemic lupus erythematosus [25, 26], multiple sclerosis [27], and Graves disease [28], revealing the immunosuppressive activity of IL-35 in different types of connective tissue diseases, which was mainly through regulating Th17 cells and regulatory T cells [29]. However, Lian et al. [30] showed that IL-35 subunits were elevated in liver tissue and were positively associated with degrees of hepatic inflammation and fibrosis in patients with autoimmune hepatitis. Furthermore, IL-35 induced expansion of myeloid-derived suppressor cells in liver microenvironment, resulting in a potential protective activity of IL-35 in autoimmune hepatitis [30]. Similarly, serum level of IL-35 was higher in psoriatic arthritis patients, which was characterized by bone erosion in peripheral joints and bone formation in spinal joints [31]. IL-35 was also upregulated at the sites of inflammation in RA synovium, which was even higher than in psoriatic arthritis synovium [32]. Moreover, IL-35 stimulated osteoblasts differentiation activated by TNF-α [33]. In turn, TNF-α induced the elevation of IL-35 secretion by PBMCs, which further promoted the release of IL-1β and IL-6 in RA patients [32], suggesting the proinflammatory potential of IL-35 in RA pathogenesis. Thus, the opposing mechanisms of anti-inflammatory and proinflammatory function of IL-35 might be related to the ultimate development of autoimmune disorder, and in a context dependent manner [20]. Our current results were consistent with the previous finding, revealing the downregulation of circulating IL-35 in PBC patients [21]. Importantly, lower plasma IL-35 was accompanied by higher ALP level in PBC patients. ALP, the representative marker for cholestasis, was localized in both canalicular and basolateral membranes and in the entire cytoplasm of bile duct epithelial cells, and was strongly increased in liver tissue compared with controls and patients with chronic hepatitis [34]. The increased expression of serum ALP might be the result of the elevation of hepatic ALP in PBC [34]. Thus, the lower level of IL-35 might serve as a marker for cholestasis in PBC. However, the role of IL-35 reduction in PBC patients still needs to be further elucidated.

CD4^+^ Th cells are involved in the pathogenesis of PBC. Th1 cytokines and chemokines, including IFN-γ, chemokine (C-X-C motif) ligand 9, and IFN-γ-induced protein-10, were increased in PBC patients and were reduced after ursodeoxycholic acid treatment [35]. Decreased liver-infiltrating CD4^+^ Th1 cells indicated a good response to ursodeoxycholic acid therapy [36]. Th9-secreting cytokine IL-9 was found in higher concentrations in peripheral blood and liver tissues of PBC patients [37]. Th17 population was decreased in the circulation and was associated with greater accumulation in the liver during PBC progression [38]. Deletion of IL-22, which was the hallmark cytokine for Th22 cells, reduced biliary injury in a PBC mouse model [39]. Herein, we found that transcription factors and cytokine productions by CD4^+^ Th cells were robustly increased in the peripheral blood of PBC patients, indicating the involvement of Th1, Th9, Th17, and Th22 cells in PBC. Peripheral CD4^+^ Th cells exhibited a stronger inflammatory phenotype in PBC, which might be important for the induction of biliary damage in PBC patients. IL-35 dampened anti-tumor activity of Th1 cells in peripheral blood and bronchoalveolar lavage fluid in patients with non-small cell lung cancer [40]. IL-35 also inhibited Th17 response in various diseases progression, including allergic rhinitis [41], visceral leishmaniasis [42], acute hepatitis B [43], etc. Our previous study also showed that IL-35 suppressed Th9 cell activity in patients with hepatocellular carcinoma [18]. However, the regulatory function of IL-35 to peripheral CD4^+^ Th cells in PBC was not fully consistent with the role in other diseases. IL-35 level did not show significant correlation with absolute count for CD4^+^ T cells. In vitro IL-35 stimulation did not affect Th1 and Th9 differentiation, but it suppressed Th17 and Th22 function in PBC patients. This finding indicated that Th17 and Th22 cells might be more sensitive to exogenous IL-35 stimulation in PBC patients and might be a key mediator for the pathogenesis of PBC. Taken together, IL-35 appeared to be an essential regulator for effector CD4^+^ T cell dysfunction in PBC.

CD8^+^ T cells mediated cytotoxicity and induced the destruction of biliary epithelial cells [44, 45]. CD8^+^ T cells revealed cytotoxicity through two independent mechanisms. On the one hand, secretion of cytotolytic molecules played vital roles in CD8^+^ T cell-induced target cell death, and this process required direct cell-to-cell contact. On the other hand, secretion of proinflammatory cytokines also contributed to cytotoxicity, which was in a cell-to-cell contact independent manner [14, 16]. Circulating CD8^+^ T cells from PBC patients expressed higher levels of perforin, granzyme B, and granulysin as well as elevated IFN-γ and TNF-α. This finding indicated that peripheral CD8^+^ T cells exhibited enhanced proinflammatory and cytotoxic phenotype during PBC. Importantly, our previous data suggested that IL-35 induced CD8^+^ T cell exhaustion in chronic viral hepatitis [12], liver cirrhosis [14], acute-on-chronic liver failure [15], and hepatocellular carcinoma [16]. Our current results revealed that there was no significant correlation between circulating IL-35 expression and CD8^+^ T cell absolute count. Importantly, in vitro IL-35 stimulation only reduced the CD8^+^ T cell-mediated target cell death in direct contact co-culture system, but not in the indirect contact co-culture system. This process was accompanied by downregulation of cytotoxic molecules and proinflammatory cytokines, as well as upregulation of immune checkpoint receptors. This finding indicated that cytotoxic molecules, but not proinflammatory cytokines, might be the predominant mediators for CD8^+^ T cells in PBC. Cytokine-induced cytotoxic might not be sufficient for cytolytic activity to HIBECs. Collectively, IL-35 stimulation suppressed non-specific CD8^+^ T cell cytotoxicity in PBC.

There were several limitations in the current study. First, we only analyzed peripheral blood but not liver-infiltrating T cell function in PBC. The phenotype of T cells might be altered in liver microenvironment in PBC patients. Second, the in vivo experiments using animal models should be performed to confirm the effect of IL-35 on T cell activity in PBC. Third, we only analyzed the regulatory function of IL-35 on T cells. Myeloid cells and other immune cells also play important roles in mediating PBC. The role of IL-35 on modulating these cells will be further elucidated.

## Conclusion

Plasma IL-35 level was reduced, whereas peripheral T cell activity was enhanced in PBC patients. Exogenous IL-35 stimulation in vitro suppressed Th17/Th22 response and inhibited cytotoxicity of CD8^+^ T cells from PBC patients. Reduced circulating IL-35 might be insufficient to suppress T cell function, leading to the immune dysregulation in PBC patients. Thus, IL-35 may be considered as a therapeutic target for PBC.

## Supplemental Data

**Figure S1.**
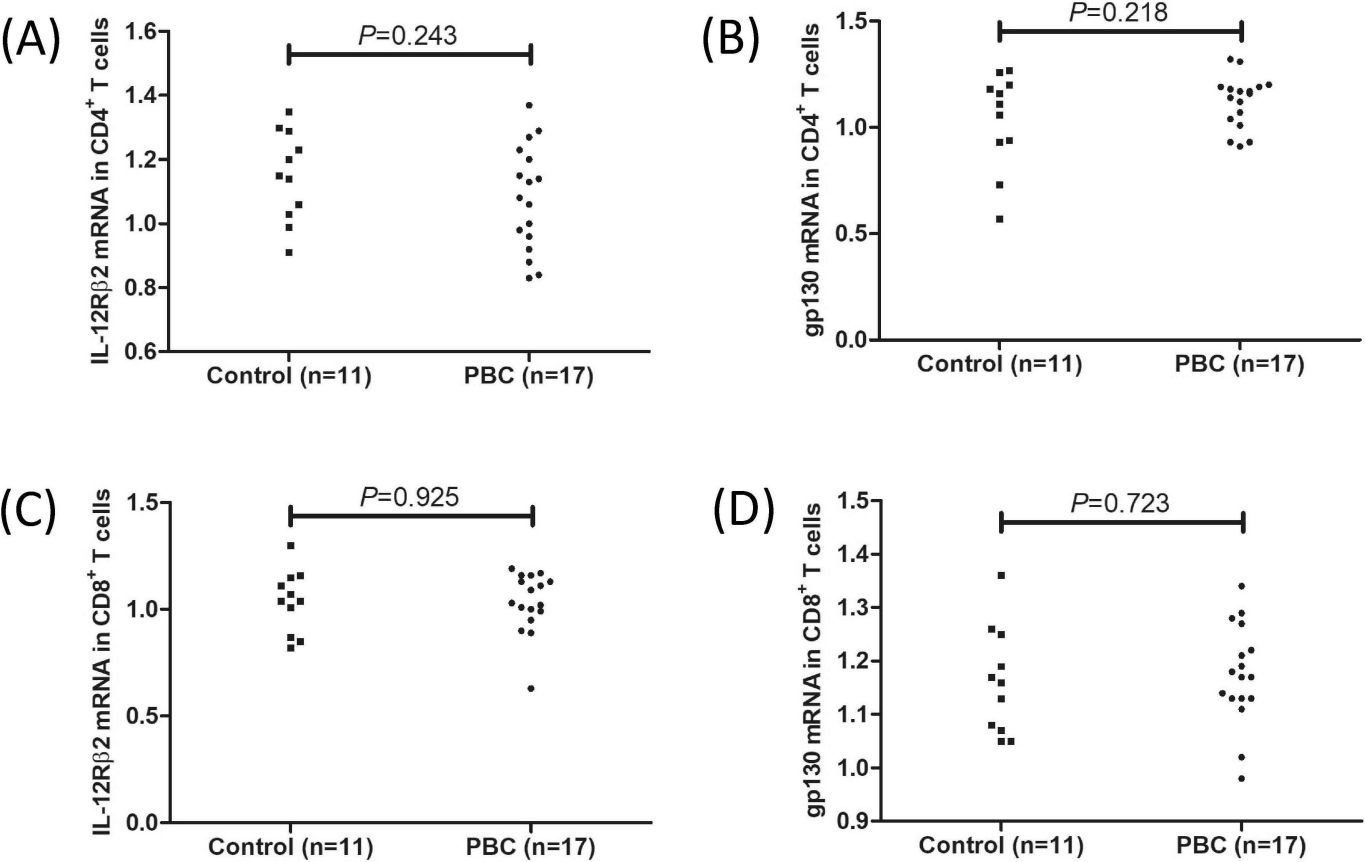
**IL-35 receptor expression in CD4^+^ and CD8^+^ T cells in controls and PBC patients.** Peripheral CD4^+^ T cells and CD8^+^ T cells were sorted from controls (*n* ═ 11) and PBC patients (*n* ═ 17). Cells were harvested for real-time RT-PCR. There were no significant differences in (A) IL-12Rß2 mRNA in CD4^+^ T cells, (B) gp130 mRNA in CD4^+^ T cells, (C) IL-12Rß2 mRNA in CD8^+^ T cells, (D) gp130 mRNA in CD8^+^ T cells between controls and PBC patients. Significance was assessed using Student’s t test. PBC: Primary biliary cholangitis; IL-12Rß2: Interleukin 12 receptor β2; RT-PCR: Reverse transcription polymerase chain reaction.

## References

[1] Tsuneyama K, Baba H, Morimoto Y, Tsunematsu T, Ogawa H (2017). Primary biliary cholangitis: its pathological characteristics and immunopathological mechanisms. J Med Invest.

[2] Gulamhusein AF, Hirschfield GM (2020). Primary biliary cholangitis: pathogenesis and therapeutic opportunities. Nat Rev Gastroenterol Hepatol.

[3] Chalifoux SL, Konyn PG, Choi G, Saab S (2017). Extrahepatic manifestations of primary biliary cholangitis. Gut Liver.

[4] Lleo A, Leung PSC, Hirschfield GM, Gershwin EM (2020). The pathogenesis of primary biliary cholangitis: a comprehensive review. Semin Liver Dis.

[5] Yong L, Chunyan W, Yan Y, Wanyu L, Huifan J, Pingwei Z (2021). Expanded circulating peripheral helper T cells in primary biliary cholangitis: Tph cells in PBC. Mol Immunol.

[6] Han Y, Bian ZH, Yang SY, Wang CB, Li L, Yang YQ (2022). Single-cell characterization of hepatic CD8^+^ T cells in a murine model of primary biliary cholangitis. Front Immunol.

[7] Niedbala W, Wei XQ, Cai B, Hueber AJ, Leung BP, McInnes IB (2007). IL-35 is a novel cytokine with therapeutic effects against collagen-induced arthritis through the expansion of regulatory T cells and suppression of Th17 cells. Eur J Immunol.

[8] Collison LW, Workman CJ, Kuo TT, Boyd K, Wang Y, Vignali KM (2007). The inhibitory cytokine IL-35 contributes to regulatory T-cell function. Nature.

[9] Collison LW, Delgoffe GM, Guy CS, Vignali KM, Chaturvedi V, Fairweather D (2012). The composition and signaling of the IL-35 receptor are unconventional. Nat Immunol.

[10] Zhang J, Zhang Y, Wang Q, Li C, Deng H, Si C (2019). Interleukin-35 in immune-related diseases: protection or destruction. Immunology.

[11] Yang L, Jia S, Shao X, Liu S, Zhang Q, Song J (2019). Interleukin-35 modulates the balance between viral specific CD4^+^CD25^+^CD127^dim/-^ regulatory T cells and T helper 17 cells in chronic hepatitis B virus infection. Virol J.

[12] Shao X, Ma J, Jia S, Yang L, Wang W, Jin Z (2017). Interleukin-35 suppresses antiviral immune response in chronic hepatitis B virus infection. Front cell Infect Microbiol.

[13] Liu S, Zhang Q, Shao X, Wang W, Zhang C, Jin Z (2017). An immunosuppressive function of interleukin-35 in chronic hepatitis C virus infection. Int Immunopharmacol.

[14] Yang L, Liu S, Zhang Q, Jia S, Qiu C, Jin Z (2022). Overexpression of ascitic interleukin-35 induces CD8^+^ T cell exhaustion in liver cirrhotic patients with spontaneous bacterial peritonitis. Int Immunopharmacol.

[15] Yang L, Zhang Q, Song J, Wang W, Jin Z (2020). Interleukin-35 suppresses CD8^+^ T cell activity in patients with viral hepatitis-induced acute-on-chronic liver failure. Dig Dis Sci.

[16] Yang L, Shao X, Jia S, Zhang Q, Jin Z (2019). Interleukin-35 dampens CD8^+^ T cells activity in patients with non-viral hepatitis-related hepatocellular carcinoma. Front Immunol.

[17] Liu S, Yang L, Jia S, Zhao R, Jin Z (2021). Interleukin-35 suppresses the activity of natural killer-like B cells in patients with hepatocellular carcinoma. Int Immunopharmacol.

[18] Zhang Q, Yang L, Liu S, Zhang M, Jin Z (2021). Interleukin-35 suppresses interleukin-9-secreting CD4^+^ T cell activity in patients with hepatitis B-related hepatocellular carcinoma. Front Immunol.

[19] Su LC, Liu XY, Huang AF, Xu WD (2018). Emerging role of IL-35 in inflammatory autoimmune diseases. AutoimmunRev.

[20] Sakkas LI, Mavropoulos A, Perricone C, Bogdanos DP (2018). IL-35: a new immunomodulator in autoimmune rheumatic diseases. Immunol Res.

[21] Li T, Huang Y, Liu P, Liu Y, Guo J, Zhang W (2018). Lower plasma levels of IL-35 in patients with primary biliary cirrhosis. Tohoku J Exp Med.

[22] Lindor KD, Gershwin ME, Poupon R, Kaplan M, Bergasa NV, Heathcote EJ (2009). Primary biliary cirrhosis. Hepatology.

[23] Sun M, Xing H (2021). Interleukin-35 regulates peripheral T cell activity in patients with Kawasaki disease. Int Immunopharmacol.

[24] Guan SY, Leng RX, Khan MI, Qureshi H, Li XP, Ye DQ (2017). Interleukin-35: a potential therapeutic agent for autoimmune diseases. Inflammation.

[25] Ouyang H, Shi YB, Liu ZC, Wang Z, Feng S, Kong SM (2014). Decreased interleukin 35 and CD4^+^EBI3^+^ T cells in patients with active systemic lupus erythematosus. Am J Med Sci.

[26] Ye Z, Jiang Y, Sun D, Zhong W, Zhao L, Jiang Z (2019). The plasma interleukin (IL)-35 level and frequency of circulating IL-35^+^ regulatory B cells are decreased in a cohort of Chinese patients with new-onset systemic lupus erythematosus. Sci Rep.

[27] Eslami M, Rafiei A, Baghbanian SM, Fattahi S, Yazdani Z, Valadan R (2022). Serum levels and genetic variation of IL-35 are associated with multiple sclerosis: a population-based case-control study. Immunol Res.

[28] Saeed MH, Kurosh K, Zahra A, Hossein DM, Davood R, Ataollahi MR (2021). Decreased serum levels of IL-27and IL-35 in patients with graves disease. Arch Endocrinol Metab.

[29] Wang D, Lei L (2021). Interleukin-35 regulates the balance of Th17 and Treg responses during the pathogenesis of connective tissue diseases. Int J Rheum Dis.

[30] Lian M, Zhang J, Zhao L, Chen X, Peng Y, Wang Q (2019). Interleukin-35 regulates immune microenvironment of autoimmune hepatitis through inducing the expansion of myeloid-derived suppressor cells. Front Immunol.

[31] Li J, Liu L, Rui W, Li X, Xuan D, Zheng S (2017). New interleukins in psoriasis and psoriatic arthritis patients: the possible roles of interleukin-33 to interleukin-38 in disease activities and bone erosions. Dermatology.

[32] Filková M, Vernerová Z, Hulejová H, Prajzlerová K, Veigl D, Pavelka K (2015). Pro-inflammatory effects of interleukin-35 in rheumatoid arthritis. Cytokine.

[33] Li Y, Yuan L, Jiang S, Liu S, Xia L, Shen H (2019). Interleukin-35 stimulates tumor necrosis factor-alpha activated osteoblasts differentiation through Wnt/beta-catenin signaling pathway in rheumatoid arthritis. Int Immunopharmacol.

[34] Suzuki N, Irie M, Iwata K, Nakane H, Yoshikane M, Koyama Y (2006). Altered expression of alkaline phosphatase (ALP) in the liver of primary biliary cirrhosis (PBC) patients. Hepatol Res.

[35] Limongi F (2015). Th1 cytokines and chemokines in primary biliary cirrhosis. Clin Ter.

[36] Yu K, Li P, Xu T, Xu J, Wang K, Chai J (2021). Decreased infiltration of CD4^+^ Th1 cells indicates a good response to ursodeoxycholic acid (UDCA) in primary biliary cholangitis. Pathol Res Pract.

[37] Wang Q, Luo D, Sun X, Feng N, Li H, Hu J (2016). [Increased expression of IL-9 in peripheral blood and liver tissues of patients with primary biliary cirrhosis]. Xi Bao Yu Fen Zi Mian Yi Xue Za Zhi.

[38] Shi T, Zhang T, Zhang L, Yang Y, Zhang H, Zhang F (2015). The distribution and the fibrotic role of elevated inflammatory Th17 cells in patients with primary biliary cirrhosis. Medicine (Baltimore).

[39] Kawata K, Tsuda M, Yang GX, Zhang W, Tanaka H, Tsuneyama K (2013). Identification of potential cytokine pathways for therapeutic intervention in murine primary biliary cirrhosis. PLoS One.

[40] Wang HM, Zhang XH, Feng MM, Qiao YJ, Ye LQ, Chen J (2018). Interleukin-35 suppresses the antitumor activity of T cells in patients with non-small cell lung cancer. Cell PhysiolBiochem.

[41] Xie F, Hu Q, Cai Q, Yao R, Ouyang S (2020). IL-35 inhibited Th17 response in children with allergic rhinitis. ORL J Otorhinolaryngol Relat Spec.

[42] Asad M, Sabur A, Kamran M, Shadab M, Das S, Ali N (2021). Effector functions of Th17 cells are regulated by IL-35 and TGF-beta in visceral leishmaniasis. FASEB J.

[43] Teng DK, Liu Y, Lv YF, Wang L, Zhang W, Wang JP (2019). Elevated interleukin-35 suppresses liver inflammation by regulation of T helper 17 cells in acute hepatitis B virus infection. Int Immunopharmacol.

[44] Zhang S, Tao X, Wang L, Chen H, Zhao L, Sun J (2022). Downregulation of programmed death-1 pathway promoting CD8 + T cell cytotoxicity in primary biliary cholangitis. Dig Dis Sci.

[45] Zhao SX, Li WC, Fu N, Zhou GD, Liu SH, Jiang LN (2020). Emperipolesis mediated by CD8^+^ T cells correlates with biliary epithelia cell injury in primary biliary cholangitis. J Cell Mol Med.

